# Preparing to React: A Behavioral Study on the Interplay between Proactive and Reactive Action Inhibition

**DOI:** 10.3390/brainsci11060680

**Published:** 2021-05-22

**Authors:** Stefania C. Ficarella, Andrea Desantis, Alexandre Zénon, Boris Burle

**Affiliations:** 1 CNRS—Le Centre National de la Recherche Scientifique, LNC, Aix Marseille University, F-13331 Marseille, France; boris.burle@univ-amu.fr; 2 The French Aerospace Lab ONERA, Département Traitement de l’Information et Systèmes, 13661 Salon-de-Provence, France; andrea.desantis@onera.fr; 3 INCC—Integrative Neuroscience & Cognition Center UMR 8002, CNRS, Université de Paris, F-75006 Paris, France; 4 Institut de Neurosciences de la Timone (UMR 7289), CNRS, Aix-Marseille Université, F-13005 Marseille, France; 5 Institut de Neuroscience Cognitive et Intégrative d’Aquitaine (UMR5287), CNRS and Université de Bordeaux, F-33076 Bordeaux, France; alexandre.zenon@u-bordeaux.fr

**Keywords:** proactive, reactive, action inhibition, inhibitory control, motor preparation, EMG, motor inhibition, compatibility effect, partial error

## Abstract

Motor preparation, based on one’s goals and expectations, allows for prompt reactions to stimulations from the environment. Proactive and reactive inhibitory mechanisms modulate this preparation and interact to allow a flexible control of responses. In this study, we investigate these two control mechanisms with an *ad hoc* cued Go/NoGo Simon paradigm in a within-subjects design, and by measuring subliminal motor activities through electromyographic recordings. Go cues instructed participants to prepare a response and wait for target onset to execute it (Go target) or inhibit it (NoGo target). Proactive inhibition keeps the prepared response in check, hence preventing false alarms. Preparing the cue-coherent effector in advance speeded up responses, even when it turned out to be the incorrect effector and reactive inhibition was needed to perform the action with the contralateral one. These results suggest that informative cues allow for the investigation of the interaction between proactive and reactive action inhibition. Partial errors’ analysis suggests that their appearance in compatible conflict-free trials depends on cue type and prior preparatory motor activity. Motor preparation plays a key role in determining whether proactive inhibition is needed to flexibly control behavior, and it should be considered when investigating proactive/reactive inhibition.

## 1. Introduction

Interacting efficiently with the environment requires making predictions, planning responses, correcting them online and, eventually, learning from errors. Predictions are based, among other things, on expectations, context interpretation, beliefs, and desires. Environmental cues, such as the lights being switched off at an opera house, inform us of upcoming events, such as the beginning of a lyrical piece. Therefore, they allow us to form predictions and plan our actions accordingly. Preparing a response in advance, however, may induce a cost, since it may lead to unwanted responding. Hence, such actions sometimes need to be withheld, either to prevent premature responses or because there is uncertainty as to whether they will ultimately have to be performed or not [1,2]. This process is called proactive action inhibition, and it is often contrasted with reactive forms of action control (triggered by environmental stimuli explicitly instructing one not to produce the intended action), usually tested in laboratory settings using Go/NoGo or Stop Signal Paradigms—SSP [3,4,5], but see [6,7] for a different account.

Proactive inhibition is a general term that refers to two main processes. First, contextual information, which can be provided by cues (e.g., [8]), may lead participants to expect that their actions might need to be withheld (“preparing to stop”). For example, studies comparing simple RT tasks for tasks requiring, in some trials, actions to be withheld, typically find slower RTs in the latter [3,9], which can be considered evidence of this aspect of proactive control. Contextual information has also been shown to impact action execution by reducing motor times of reaching movements, suggesting an interaction between proactive inhibition and online control of actions [9].

Another form of proactive inhibition, often termed “impulse control” [10,11], relates to the prevention of premature responses. Many studies reported that, when preparing an action, while cortical excitability of the primary motor cortex increases, the cortico-spinal excitability concomitantly decreases, reflecting an active inhibition [12,13] whose strength covaries with performance [14]. This inhibition has been interpreted as a process to prevent premature responses when the excitability of the motor structures is close to the one triggering a response. It can be construed as a top-down suppressive inhibition that is activated whenever actions are being planned at cortical and subcortical levels to prevent an early execution of such responses. Proactive inhibition as impulse control tries to balance motor preparation mechanisms, resulting in variable cortico-spinal excitability levels [12,13,14,15,16,17,18]; see [19] for a review. Although this proactive inhibition mechanism tries to prevent muscle activation from triggering a response, “leaking” preparatory motor activity, resulting from an imbalance between activation and inhibition mechanisms, can be detected as peripheral EMG activity. Peripheral (muscular level) markers of motor preparation lie on a continuum from an absent one to a full, peripherally detectable, activity preceding an action’s execution. While absent peripheral muscular activity may result from efficient impulse control, and also from a mere absence of response preparation, strong EMG activity is likely the result of robust motor preparation that is barely kept under control by such proactive mechanisms. As claimed elsewhere [20,21], very few action inhibition studies have tested for the presence of ongoing motor preparation activities, assuming that participants prepare the instructed response before target onset. In this study, we used EMG to assess whether motor preparation was present before target onset (see Section 2 for details).

While the interplay between proactive inhibition and motor preparation is complex, the latter is recognized as the critical factor disentangling a (pro)active inhibitory process from a strategic delay in motor planning [19,20,21,22]. As a matter of fact, planned actions that need to be withheld for a fixed, short duration might not necessarily rely on such “impulse control” mechanisms [20]. Indeed, “impulse control” cannot exist without motor preparation (the impulse itself). Therefore, the choice of the task is pivotal to induce participants to prepare responses in advance, and ultimately to test the effects of proactive and reactive inhibition on performance.

Classical versions of the “Stop Signal Paradigm” (SSP) require action cancellation following a stop signal, which represents the prototypical form of reactive inhibition. In contrast, the nature of the target on Go/NoGo paradigms immediately requires participants to respond or withhold a (supposedly) prepared action; this is proactive inhibition-modulated action restraint [23,24,25,26]. Modified versions of the SSP and Go/NoGo task, using informative cue instructing participants either that a stop (or NoGo) signal might occur [8,27] or that some or all effectors might need to be inhibited [4,5,28], have been adopted to investigate the effects of proactive control. Some of these studies, however, adopted a blocked design in which participants know the cue identity that will be used throughout all the trials of the block. It is well known that block designs reduce the impact of cues on preparatory mechanisms. The uncertainty as to whether a response might need to be withheld (reduced in block designs) is indeed a major factor influencing both proactive inhibition and motor preparation [3,9,20,22,29,30].

These preparatory processes (activation and inhibition) interact with an everchanging (internal and external) environment, in which both relevant and irrelevant information tend to activate responses for fast reactions. Consequently, the activated response is sometimes incorrect, behavioral errors being an index of such incorrect response activations. Detailed analysis of response execution dynamics on such errors revealed (failed) attempts to stop this incorrect response, evidencing an online control of actions [31,32]. Overt behavioral errors are only the tip of the iceberg, however. Electromyographic (EMG) recordings in such tasks reveal, on some behaviorally correct trials, subliminal incorrectly activated responses [33,34,35], termed “partial errors”. Such partial errors that largely remain unconscious [36,37] are of particular interest for the study of online control: they clearly indicate that the incorrect response was activated and later inhibited while a corrective response is being activated. They are often studied in so-called conflict tasks, such as the Simon task [38], in which a visuospatial conflict is generated between the task-irrelevant lateralization of the presented stimulus and the side of the required response based on an orthogonal dimension of the stimulus, such as its color (e.g., respond “left” for a red stimulus that can be presented on the left—compatible trial—or on the right—incompatible trial). In incompatible trials, a stimulus’ position automatically activates the (incorrect) ipsilateral response, resulting in more frequent errors, partial errors, and slower reaction times (RTs) than in correct trials. While finding more numerous partial errors on incompatible trials is consistent with an automatic position-based activation, their occurrence can also be affected by other variables, as suggested by the presence of partial errors on compatible trials, where automatic activation by the stimulus cannot be invoked. Their origin, which remains to be clarified, might need to be sought in the period preceding the stimulus, in which no conflicting information is present and, more precisely, in the preparatory motor activities.

In the present study, we aim at investigating proactive and reactive action inhibition mechanisms while measuring motor preparation activity through EMG, using a cued Go/NoGo Simon paradigm in a within-subjects experimental design. In each trial, an informative cue was presented before either a (more frequent) Go or NoGo lateralized target, allowing us, on the one hand, to investigate cueing effects on motor preparation and proactive inhibition mechanisms and, on the other hand, to explore conflict effects, resulting from both cue validity and stimulus-response compatibility, on reactive inhibition. The Go/NoGo nature of the task instructed participants that they might need to withhold actions, inducing a proactive “preparing to stop” mechanism that interacted, in each trial, with the information carried by the cue. Following a NoGo cue, participants were informed that they would most likely not have to execute a response. We hypothesized that such a context would lead participants to increase their proactive inhibition as a “preparing to stop” process. Under such conditions, slower RTs can be hypothesized in (unexpected) NoGo cue Go trials. Conversely, Go cues instructed participants that they will most likely have to respond. Coherently, participants prepared a response, lowering the threshold for their execution but withholding them for the duration of the foreperiod through proactive impulse control mechanisms (to which we will refer to in the manuscript as proactive inhibition). Up to target onset (whose identity determines cue validity), valid and invalid (Go) cue trials are equal in terms of proactive inhibition demands, allowing us to isolate reactive inhibition components. We will interpret the results in light of the activation–suppression model [39], showing that motor preparation plays a pivotal role in shaping the relationship between proactive and reactive inhibition.

## 2. Materials and Methods

### 2.1. Participants

Eighteen volunteers (mean age 23 ± 3 years old, 6 males, 1 left-handed) with normal or corrected-to-normal vision (a priori inclusion criteria) participated in the study after giving written informed consent. All participants reported no psychological and neurological disorders (a priori inclusion criteria). The study was approved by the “Comité de Protection des Personnes Sud Méditerranée 1” (agreement n° [10–41]). Data from two participants had to be excluded from the analyses due to a trigger malfunction and noisy EMG data, respectively. Analyses were therefore conducted on data from 16 participants.

### 2.2. Stimuli and Procedure

Participants were seated in a comfortable chair placed inside of a Faraday cage throughout the duration of the experiment. White stimuli were displayed on a black background of a CRT monitor (85 Hz refresh rate) placed 1 m away from participants’ eyes, and responses were collected through two buttons placed 20 cm apart on a table in front of the subjects. Participants held the cylindrically shaped hand-grips, fixed on the table, throughout the experiment, resting their thumbs on the upper part, where the button was placed. All of the stimuli and responses were controlled by a PC running a custom-made script written in the Python language through OpenSesame software [40]. We designed a cued Go/NoGo version of the Simon task [38], detailed below. Participants performed 72 practice trials and 960 experimental trials, for a total duration of one hour and a half, with small breaks every 5 min. A fixation cross was presented on the center of the screen throughout the whole experiment. The inter-trial interval (ITI) was a variable between 1700 and 2500 ms, after which one of three possible cues, randomly selected, was superimposed on the fixation cross for 300 ms. The three cues were: an empty isosceles triangle (with each side measuring 4.3 cm, a base of 4.7 cm, and 3.5 cm height), an empty circle (3.5 cm in diameter), and an empty square (each side 3.5 cm long). The same shapes filled in white were used as targets. Targets appeared 600 ms after cue offset at one of two equiprobable locations randomly selected for each trial: to the left or to the right of the fixation cross (the center of each shape was 8 cm away from the fixation cross). Targets remained visible for 1000 ms, during which responses were collected.

Participants were asked to press, as fast as possible, the left or right button as a function of the target. Half of the participants (pseudo-randomly selected) were requested to press the left button when the target was a square, the right button when the target was a triangle, and not to respond if the target was a circle. For the other half of the participants, the instructions were inverted between the square and the triangle, but the circle was always a No-Go stimulus. We chose not to counterbalance all possible target-response associations because, while squares and triangles have at least two angles pointing towards the left and the right, the circle has no angles and it is, in this sense, different from the other shapes, making it easier for participants to identify it as a No-Go stimulus. Although irrelevant for the task at hand, a stimulus’ position (left or right) on Go (square or triangle target) trials allows us to distinguish compatible and incompatible trials, depending on whether the stimulus’ location was ipsi (e.g., a square presented on the left when participants had to press the left buttons whenever the target was a square) or contra-lateral (e.g., a square presented on the right for the same participants) to the required response.

**Figure 1:**
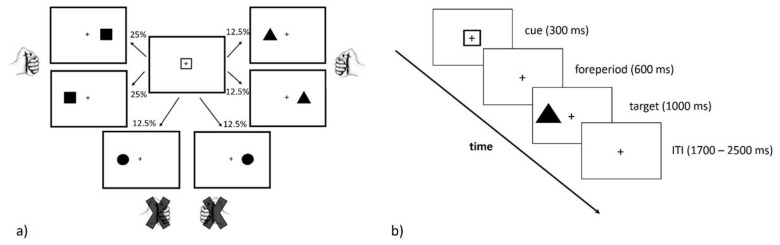
A visual representation of cue-target probabilities is displayed in (**a**). In this example, a squared central cue instructs participants to prepare a left-hand response. In 50% of the trials, a lateralized squared target was presented, requiring a left-hand button press. Such trials are termed valid cue Go trials. Conversely, a triangle target (presented in 25% of square cue trials, equiprobably on both side) required a contralateral (right-hand, in this example) button press, representing invalid cue Go trials. For both valid and invalid trials, the Go target can be presented ipsilaterally (compatible trials) or contralaterally (incompatible trials) to the required response. Finally, a NoGo circle target (presented in 25% of square cue trials, equiprobably on both side) requires for all participants to not respond. The central cue (empty square, triangle or circle) is presented for 300 ms, followed by a foreperiod of 600 ms. An example of invalid cue incompatible trial of the cued Go/NoGo Simon paradigm is shown in (**b**). In this example, the square instructs (half of the) participants to prepare a left-hand button press. Following a 600 ms-long foreperiod, a lateralized filled-shape target is displayed for 1000 ms, during which responses are collected. A stimulus’ location can be compatible or incompatible with the side of the required response (in this case, a triangle requires a right-hand response, but it is presented on the left side). Following a variable inter-trial-interval (ITI), a new trial starts. Colors are inverted for displaying purposes.

Each cue had a 50% chance to be followed by the same shape as the target (in which case, the cue is named valid; for example, an empty square followed by filled square), and a 50% chance to be followed by one of the other two targets, equally represented in the task. The cue informed participants of the target’s likely identity and allowed them to prepare the response in advance. Despite the cue being valid in half of the trials, each alternative occurred in 25% of the trials, making the cue still informative (see Figure 1a for a visual representation of cue-target probabilities). Targets could hence match (valid trial) or not match (invalid trial) the cue, and signal either a Go (for triangle or square targets) or NoGo (circle target) trial. While Go trials preceded by a NoGo cue can be considered invalid (e.g., circle cue followed by a triangle target), we chose to indicate them as NoGo cue trials in order to distinguish them more easily from Go cue invalid trials (e.g., square cue followed by a triangle target).

Participants were instructed to trust cues and “use the information of the cue to be ready to respond as fast as possible to the target”. In fact, even if the maximum time to respond was 1 s, speed was stressed. An example of invalid cue incompatible trial structure is presented in Figure 1b. Examples of valid and NoGo cue trials can be found in the Supplementary Materials (Figure S1).

### 2.3. EMG Recordings

A continuous electromyogram (EMG) was recorded from the flexor pollicis brevis, bilaterally, with two surface active-two (Biosemi, Amsterdam, The Netherlands) Ag–AgCl electrodes, placed approximately 2 cm apart on the thenar eminences. The EMG signal was continuously monitored by the experimenter, who instructed participants to relax the muscles whenever EMG activity was detected between trials or tonic muscular activity was too high in the cue-target period (henceforth called foreperiod).

### 2.4. EMG Signal Processing

Raw data were acquired at a 2048 Hz sampling rate and offline high-pass filtered with a 10 Hz frequency cut-off. The EMG signal was processed with BrainAnalyzer (BrainProducts, Munich, Germany) and with custom scripts written in MATLAB (The Mathworks, Natick, MA, USA) or Python (www.python.org (accessed on 13 November 2020)). The onset of any EMG activity was automatically detected by a custom Python script based on Hodges and Bui (1996s) variance ratio algorithm [41] and on an ‘‘integrated profile” algorithm [42,43] (An open-source version of this Python program will soon be released, and is already accessible upon request). Visual inspection (1) ensured that there was at most one marked EMG activity per channel in the foreperiod, and (2) allowed us to visually correct some inaccuracies in the algorithm-generated markers’ placement. The experimenter performing EMG visual inspection was not informed of the cue type and performance (correct vs. error), in order to avoid any implicit bias. Whenever more than one EMG burst occurred in the foreperiod, the earliest motor activity was taken into account. Trials in which at least one EMG burst could be detected during the foreperiod were considered as containing motor preparation activity. Although activation at the cortical level could be present in trials without preparatory EMG activity, this approach is a conservative one: while trials containing peripherally (EMG)-detectable motor preparation activity can be certainly categorized as presenting a motor preparation, the remaining trials may still contain preparatory activation at the cortical level. This should reduce potential differences between the two trial types. Should we nonetheless find any difference between the two EMG-based categories, we could therefore ascribe this (likely underestimated) effect to motor preparation. Based on the EMG activity pattern detected following Go target onset, trials were categorized into three types: when only one EMG activity was detected on the correct side, the trials was categorized as pure correct (PC); when the correct response (and EMG) was preceded by a subliminal EMG activity on the incorrect side (a partial error), the trial was categorized as incorrect-correct (IC). Finally, when an incorrect EMG activity resulted in an incorrect response, the trial was an error (Err). In PC trials, only one EMG activity was detected on the correct side, whereas IC trials were composed of an incorrect subthreshold EMG activation, followed by a corrective response. Finally, error trials included only one incorrect EMG activity performed before the 1000 ms time limit. Trials not entered into one of these categories were judged too few for a proper analysis and were discarded.

### 2.5. Data Analysis

Statistical analyses were performed using SPSS software (IBM Corp. Released 2020. IBM SPSS Statistics for Windows, Version 27.0. Armonk, NY, USA). Whenever possible, repeated measure ANOVAs were used to test the effects of the different factors on the variable of interest. To this aim, normality of the inter-participant data was assessed with the Kolmogorov–Smirnov test. Whenever Mauchly’s sphericity test was statistically significant, the Greenhouse–Geisser correction was applied. Results are reported with the uncorrected degrees of freedom along with the Greenhouse–Geisser epsilon (ε, [44]). The effect size estimate (partial eta squared—η^2^—for parametric ANOVAs and Kendall’s W for Friedman tests) is reported for all main analyses. Bonferroni post hoc correction was applied to multiple pairwise comparisons. Alternatively, planned comparisons were performed using paired samples Student’s *t*-tests. The latter was adopted whenever comparisons were strongly hypothesis-driven or when the number of participants was not equal among task conditions (e.g., absence of partial errors for some cue conditions), therefore not allowing a full design ANOVA. For data that did not respect the assumptions for parametrical tests, non-parametric ANOVA (Friedman test) for dependent samples with Wilcoxon post hoc test was performed instead. Correlations were run using Pearson’s test. For all analyses, the significant level was set to α = 0.05.

To analyze the cueing effect (valid, invalid and NoGo cue) on the evolution of the compatibility effect (incompatible RTs—compatible RTs) across the RT distribution, RTs stemming from correct trials only (PCs and ICs) were “vincentized” [45,46,47], using a customized Python script. RTs from each participant, separately for each cue and compatibility condition, were first ranked in ascending order and then binned into 5 classes (quintiles) of equal trial size. The mean of each quintile was then obtained, before calculating the average compatibility effect for each subject, bin, and cue condition. This average was plotted against mean RTs in the so-called delta-plot [39,48,49]. To compute the individual Conditional Accuracy Function (CAF), we recalculated the vincentized RTs, including both incorrect and correct responses. The percentage of correct responses for each quintile was then plotted against average RTs of each quintile. Finally, while correct responses include both pure correct (PC) and partial errors (IC), a modified Conditional Activation Function [50,51] was computed. In this analysis, the first EMG activity of each trial was taken into account, noting them as correct for PC trials and as incorrect for partial and full errors. We then plotted the percentage of first correct EMG activations for each quintile against average RTs (Conditional incorrect activation function—CIAF).

## 3. Results

### 3.1. Mean Performance

#### 3.1.1. Reaction Times (RTs)

When the target was a circle (NoGo trial), participants were instructed to not respond, likely involving (global) reactive inhibition to prevent the execution of any response. False alarms to NoGo targets were very rare (average 2.4 ± 2%), and therefore not analyzed. However, it is interesting to note that, before all false alarms, an EMG activity was detected in the foreperiod.

Therefore, all subsequent analyses were performed in Go trials only. In this first analysis, we checked whether cue type (valid, invalid, NoGo) and compatibility (compatible vs. incompatible) effects could be found on average RTs and accuracy (percentage of correct responses). We expected to replicate previous findings on the compatibility effect, with incompatible trials inducing slower RTs and more errors. As for cues, we expected to find a positive effect of valid cues (faster RTs and less errors) and a negative effect of invalid cues (slower RTs and more errors). Proactive inhibition, considered as a “preparing to stop” mechanism, is expected to be higher in NoGo cue trials, slowing down RTs in NoGo cue Go trials.

**Figure 2:**
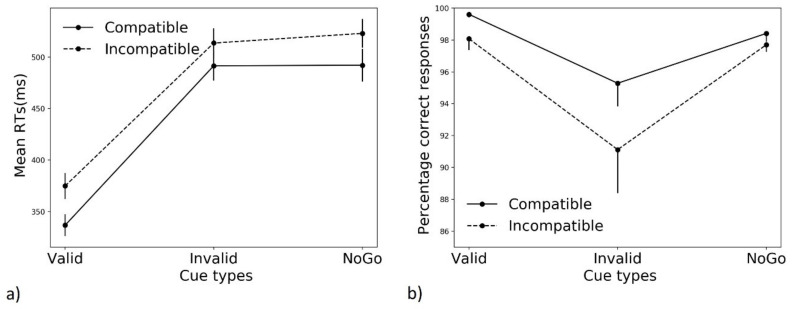
Average RTs (**a**) and percentage of correct responses (**b**) are displayed separately for compatible (filled line) and incompatible (dashed line) trials, and for each cueing condition. Error bars represent the standard error.

For correct response RTs, a repeated measures ANOVA was used (factors: Cue type and Compatibility). Both factors yielded significant effects (Cue type: F (2, 30) = 124.869, ε = 0.592, *p* < 0.001, η^2^ = 0.893; compatibility: F (1, 15) = 56.958, *p* < 0.001, η^2^ = 0.792), with no significant interaction between the two (F (2, 30) = 1.660, *p* = 0.207, η^2^ = 0.1). As expected, average RTs were significantly faster for compatible than incompatible trials (*p* < 0.001, see Figure 2a). Post hoc comparisons with Bonferroni correction showed that, as predicted, valid cue trials induced faster responses with respect to invalid and NoGo cue trials (*p* < 0.001 for both cases), while there was no significant difference between invalid and NoGo cue trials (*p* = 1).

#### 3.1.2. Accuracy

Commission errors were defined as the execution (within the allotted time) of the incorrect response to a Go target (correct late responses were not included in this analysis). Accuracy was significantly modulated by the cue condition, as suggested by a non-parametric ANOVA (Friedman) on the percentage of errors generated in the three cue type conditions (main effect of cue: F (2, 16) = 14.035, *p* < 0.001, W = 0.439). The average percentage of committed errors was: 1.15 ± 1.6%, 6.78 ± 8,3%, and 1.94 ± 1.3% for valid cue, for invalid cue, and for NoGo cue trials, respectively. Planned comparisons against the control NoGo cue condition were conducted using the non-parametric Wilcoxon test: valid vs. Nogo (Z = −2.166, *p* = 0.03), invalid vs. Nogo (Z = 2.605, *p* = 0.009). These results suggest that accuracy was highest in valid cue trials (positive cueing effect) and lowest in invalid cue trials (negative cueing effect). To investigate the compatibility effect on errors for each cue type, a Wilcoxon test was conducted comparing the percentage of correct responses performed in compatible vs. incompatible trials: while a compatibility effect was present for both valid (Z = −2.395, *p* = 0.017) and invalid (Z = −2.731, *p* = 0.006) cues, it disappeared following NoGo cues (Z = −1.434, *p* = 0.15). Results suggest that incompatible trials, compared to compatible trials, elicited more errors following valid and invalid cues, but not NoGo cues (Figure 2b). We confirmed the expected results on both cueing and compatibility effects for valid and invalid cues, while the compatibility effect was absent in NoGo cue trials, when average accuracy was considered.

### 3.2. Distribution Analyses

#### 3.2.1. RTs

A similar average compatibility effect (non-significant interaction effect between cue type and compatibility factors) was found for the three cueing conditions, which may suggest that, while cueing has a main effect on overall speed, it does not modulate the compatibility effect. Distributional analyses, in contrast, revealed a large difference hidden in the mean RTs. Previous studies reported that, in the Simon task, the compatibility effect decreases as RTs increase, as evidenced by negative-going delta plots [33,45,49]. The dynamic evolution of the compatibility effect as a function of RTs has proved to be efficient in revealing relevant between-task differences [48], along with a modulation of within task dynamics [33,39].

**Figure 3:**
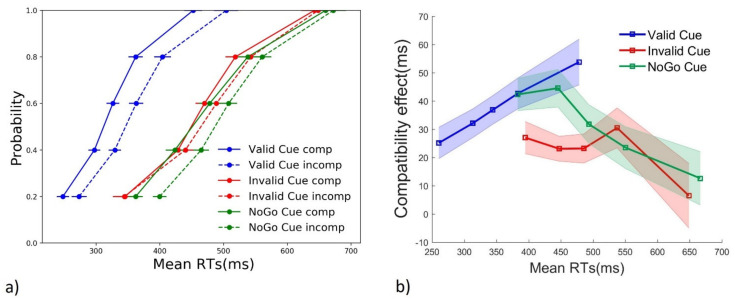
Cumulative distribution function of correct binned RTs (**a**). Delta plots calculated over correct trials (**b**). The compatibility effect (incompatible–compatible) is plotted as a function of RTs separately for each cue condition. Horizontal error bars (**a**) and shaded areas (**b**) represent the standard error.

We tested the effect of cue type on the dynamics of the compatibility effect, thanks to a repeated measures ANOVA with factors cue type (valid, invalid, NoGo) and RT bins (1 to 5). A marginal main effect for cue type (F (2, 30) = 2.940, *p* = 0.068, η^2^ = 0.164) and no main effect of bin (F (4, 60) = 1.814, *p* = 0.17, ε =0.593, η^2^ = 0.108) were observed. These two factors, however, interact significantly (F (8, 120) = 6.327, *p* = 0.002, ε = 0.315, η^2^ = 0.297). Results are displayed in Figure 3a. To investigate the interaction effect, we ran a repeated measures ANOVA separately for each cue type, comparing the compatibility effect across RT bins. We found a significant main effect of RT bins in all three types of trial (valid cue: (F (4, 60) = 7.321, *p* = 0.005, ε = 0.414, η^2^ = 0.328); invalid cue: (F (4, 60) = 3.616, *p* = 0.043, ε = 0.463, η^2^ = 0.194); NoGo cue: (F (4, 60) = 5.035, *p* = 0.013, ε = 0.507, η^2^ = 0251). Importantly, as the delta plot presented in Figure 3b shows, the direction of the effect is opposite in valid cue trials, compared to the other two conditions. Finally, since average RTs between invalid and NoGo cue trials were similar, an RT bin-by-bin pairwise comparison of the compatibility effect elicited across the two cueing conditions was conducted using paired Student’s *t*-tests. Significant differences were found for RT bins 1 (t (15) = −2.257, *p* = 0.039) and 2 (t (15) = −2.857, *p* = 0.012), in which the compatibility effect was smaller in the invalid cue, compared to the control NoGo cue condition.

To sum up, in NoGo cue trials, the compatibility effect briefly increases before sharply decreasing as RTs increase, with the negative-going slope being quite steep (Figure 3b). In invalid cue trials, this pattern is altered, with the compatibility effect for fast responses being much smaller (also see Supplementary Figure S2). Finally, this pattern was completely reversed in valid cue trials, in which a monotonic, linear relationship between the compatibility effect and RTs was found.

#### 3.2.2. Accuracy

**Figure 4:**
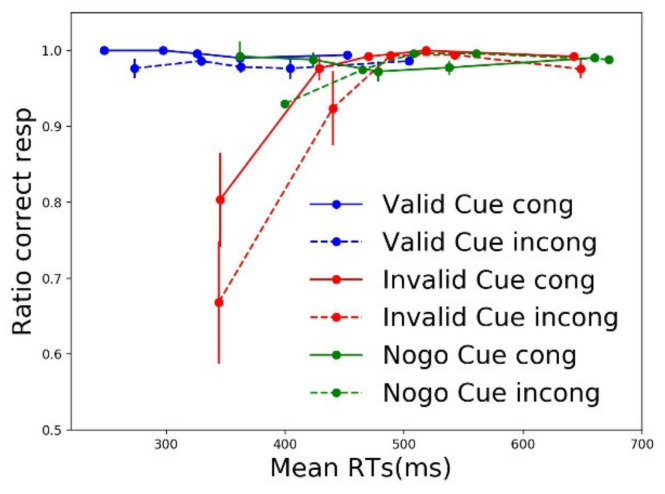
Conditional accuracy function. The ratio of correct responses is plotted as a function of RTs, divided in quintiles separately for each cue and compatibility condition. Error bars represent the standard error.

Distribution analyses were also performed for accuracy through the so-called “Conditional Accuracy function” (CAF) that allows for the exploration of the dynamic relationship between accuracy and RTs. A drop in accuracy is classically observed in incompatible trials for the fastest RTs. Such a pattern is expected for NoGo cue and, possibly, valid cue trials. Since the incorrect effector had already been primed by invalid cues, an accuracy drop for fast responses could be expected in both compatible and incompatible invalid cue trials. The CAF for the three cueing conditions, and for compatible and incompatible trials, is shown in Figure 4. Since a full ANOVA could not be conducted on percentage data, we aimed at testing the hypothesis of an accuracy drop at the fastest RT bin, using a Wilcoxon test. We found a significant difference in accuracy at the fastest RT bin for NoGo cue (W = −2.301, *p* = 0.02) and invalid cues (W = −2.970, *p* = 0.003), with a marginal effect for valid cues (W = −1.826, *p* = 0.068). In line with previous studies, incompatible conflict-inducing trials generate an automatic capture of the incorrect response that is more likely to result in an error, compared to compatible trials (NoGo cue trials: first point of solid vs. dashed green line of Figure 4). This small but significant drop in accuracy at the fastest RTs suggests that the proactive inhibition likely active on such trials was not able to counteract the automatic stimulus-driven activation of the incorrect effector, resulting in fast errors. Conversely, following valid cues, participants could keep a very high accuracy rate (first point of solid vs. dashed blue line of Figure 4), suggesting a (almost) total absence of automatic response activation by the stimulus positions (but see below). Finally, as expected, we found a very strong drop in accuracy in invalid cue trials at the faster RT bin in incompatible trials (first point of dashed red line of Figure 4). Importantly, a strong drop in accuracy (although smaller than on incompatible trials) was also observed in invalid cue compatible trials (first point of solid red line of Figure 4), and even more so in incompatible trials (first point of solid vs. dashed red line of Figure 4).

### 3.3. EMG Analyses

Distribution analyses allowed us to better reveal the dynamics of response activation, but they are limited by overt behavior. EMG analyses will allow us to track subliminal response activations whose amplitude was too low to trigger an overt response. We will first analyze such partial activations during the foreperiod to assess the impact of the information provided by the cue on motor preparation. We will next assess response activation triggered by the target and, finally, study the interaction between the two (cue-triggered and target-triggered activation).

#### 3.3.1. Motor Preparation

Participants were instructed to plan responses according to cue type and respond as quickly as possibly upon target onset. EMG activity could be detected in the foreperiod (23.8% on average), reflecting motor preparation following cue onset. First, if participants followed cue instructions, such cue-triggered EMG, activity should appear more often on the hand, indicated by the cue, and it should be very rare following NoGo cues. As results show (see Supplementary Figure S3), preparatory EMG activity in Go (square and triangle) cue trials occurred much more often on the hand corresponding to the cue (29%) than on the opposite hand (1.48%). Furthermore, participants very rarely prepared a response following a NoGo cue (3.88%). These data suggest that participants followed task instructions.

To test the effects of motor preparation on task performance, we compared trials in which a cue-triggered EMG activity was detected in the foreperiod (motor preparation trials) to trials in which no such activity could be evidenced. Since the NoGo cue instructed participants to not prepare any response, which indeed occurred very rarely (see Supplementary Figure S3), NoGo cue trials were not included in the analysis. To test the impact of motor preparation on RTs (correct responses), we compared, for each cue type (valid, invalid), mean RTs calculated on trials with vs. without overt motor preparation, with paired Student’s *t*-tests. First, for valid cues, results showed a positive effect of motor preparation on RTs (t (15) = −3.207 *p* = 0.006). Specifically, when participants prepared the response in the valid cue-instructed effector, responses to the target were faster (340 ± 56 ms) than when no over motor preparation was detected in the EMG trace (365 ± 42 ms). Motor preparation of the correct response in the foreperiod is beneficial to task performance on valid cue trials.

To test whether only the presence or also the amplitude of motor preparation activity would influence behavior on valid cue trials, a correlational analysis was run between the cumulative amplitude of cue-triggered EMG activity measured in the foreperiod and RTs. A significant negative correlation was found (Pearson coefficient R = −0.57, *p* = 0.02). Across participants, the higher the correct preparatory EMG activity, the shorter the RTs (Supplementary Figure S4).

Second, in invalid cue trials, one might expect a cost of motor preparation since, in these cases, the incorrect response is being prepared. Although results are numerically going into that direction (507, 87 ± 60 ms and 502, 3 ± 66 ms for trials with and without preparatory EMG activity, respectively), the effect was far from significant (t (15) = 0.575, *p* = 0.57). However, these results arise from average correct RTs that include both pure correct and partial error trials. An analysis that differentiates between the two could provide interesting results (see below).

The effects of motor preparation on accuracy were much less clear. While a trend for an increase in the accuracy of valid trials with motor preparation was observed (W = −1.784, *p* = 0.074), the opposite pattern in invalid cue trials was far from significant (W = −0.157, *p* = 0.875). However, when the latency of the motor preparation was considered, we found a significant positive correlation with accuracy in invalid cue trials (Pearson coefficient R = 0.61, *p* = 0.011, see Supplementary Figure S5), while it was non-significant in valid cue trials (Pearson coefficient R = 0.12, *p* = 0.66) in which a ceiling effect on accuracy likely prevented this effect arising. Since motor preparation latency was calculated with respect to target onset, bigger values indicate an earlier motor preparation activity. Therefore, the positive correlation in invalid cue trials indicates that participants who prepared the incorrectly cued effector earlier in the foreperiod had higher accuracy.

#### 3.3.2. RTs Based on EMG Categories

Trials were divided into pure correct (PC), partial errors (incorrect-correct: IC), and errors (Err). A repeated measures ANOVA with Bonferroni post hoc correction was used to test the effect of trial type on mean RTs. One subject had to be excluded from this analysis since he/she did not produce any errors. Results showed a main effect of trial type (F (2, 28) = 56.112, *p* < 0.001, ε = 0.595, η^2^ = 0.8), and planned comparisons against the control PC conditions were significant (PC vs. IC: *p* < 0.001; PC vs. Err: *p* = 0.023). In line with previous studies, partial errors induced slower responses (528 ± 52 ms), while errors induced faster responses (338 ± 81 ms) compared to PC trials (402 ± 43).

#### 3.3.3. Partial Errors’ Frequency

**Figure 5:**
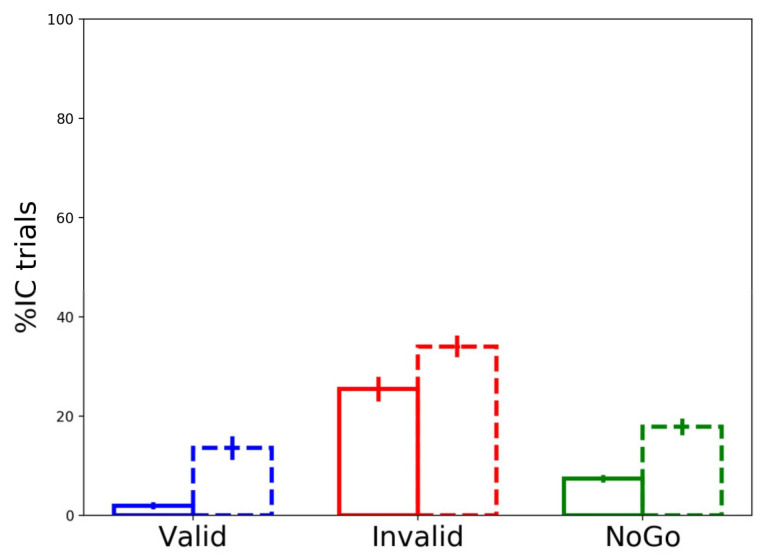
The percentage of partial errors committed following valid (blue bars), invalid (red bars), and NoGo (green bars) cues is displayed separately for compatible (filled lines) and incompatible (dashed lines) trials. Error bars represent the standard error.

Replicating previous results, partial errors were committed significantly more frequently in incompatible (65 ± 9%) than compatible (34.7 ± 9%) trials (W = −3.516, *p* < 0.001). More interestingly, the frequency of partial errors was impacted by cue type, irrespective of trial compatibility. We calculated relative frequencies of IC trials separately for each cue type over the total number of committed partial errors. Group averages of committed partial errors were 15.4 ± 11% (15 ± 20 trials), 59.4 ± 16% (51 ± 28 trials), and 25.1 ± 7% (22 ± 14 trials) for valid, invalid, and NoGo cues, respectively (see Figure 5). A non-parametric ANOVA (Friedman) was used to test the effect of cue type on the percentage of partial errors. Results showed a significant main effect (F (2, 16) = 21.125, *p* < 0.001, W = 0.660), and planned pairwise comparisons conducted with the Wilcoxon test were run to compare valid and invalid cue trials against the control NoGo cue condition. We found significant differences for both valid (W = −2.782, *p* = 0.005) and invalid (W = −3.361, *p* = 0.001) cues. Compared to NoGo cue trials, participants committed significantly fewer partial errors in valid cue trials, and significantly more partial errors in invalid cue trials.

Since partial errors occur more often in incompatible trials and their overall frequency is dependent on cue type, we tested whether the rarer ICs occurring in compatible trials were equally distributed across cue types. Figure 5 shows the relative contribution of compatible (filled lines) and incompatible (dashed lines) trials to the overall percentage of IC trials following the three cue types. In this analysis, we focused on ICs elicited in compatible trials only. To this aim, we calculated, separately for each cue type, the percentage of partial errors occurring in compatible trials (over the total IC trials occurring in compatible + incompatible trials). A non-parametric (Friedman) ANOVA was used to test the effect of cue type over percentages of compatible ICs (F (2, 16) = 26.656, *p* < 0.001, W = 0.833). Of the total of NoGo cue partial errors (filled plus dashed green lines in Figure 5), 30.3 ± 11% occurred in compatible trials (filled green line in Figure 5). Post hoc pairwise comparisons (Wilcoxon test) comparing the control NoGo condition to the other two confirmed that: in valid cue trials, partial errors were committed even less often in compatible trials (9.4 ± 12% of valid cue partial errors, Z = −3.170, *p* = 0.002; comparison blue filled line vs. green filled line in Figure 5); and, for invalid cue trials, partial errors were committed more often in compatible trials compared to the NoGo condition (42 ± 8% of invalid cue partial errors, Z = −3.124, *p* = 0.002; comparison red filled line vs. green filled line in Figure 5). These results suggest that partial errors are not only the result of lateralized targets, likely automatically triggering the ipsilateral effector, but are also generated because of motor preparation activities preceding target presentation. In line with our previous CAF results, invalid cue compatible trials not only elicited a high number of fast errors (red lines in Figure 4) but also frequent partial errors (red lines in Figure 5). More than half (57 ± 31%) of such invalid cue compatible IC trials contained cue-triggered preparatory EMG activity. While partial errors occurring in compatible trials following invalid cues can be explained by cue-triggered (incorrect) motor preparation, one may wonder why ICs occur in compatible trials following NoGo cues, and even more so after valid cues. It is interesting to note that all valid cue-compatible IC trials and 91 ± 12% of NoGo cue-compatible IC trials did not contain any preparatory EMG activity. We suggest that such rare partial errors result from lapses of attention or incorrect expectations resulting from previous trials.

#### 3.3.4. EMG-Based Accuracy Dynamics

**Figure 6:**
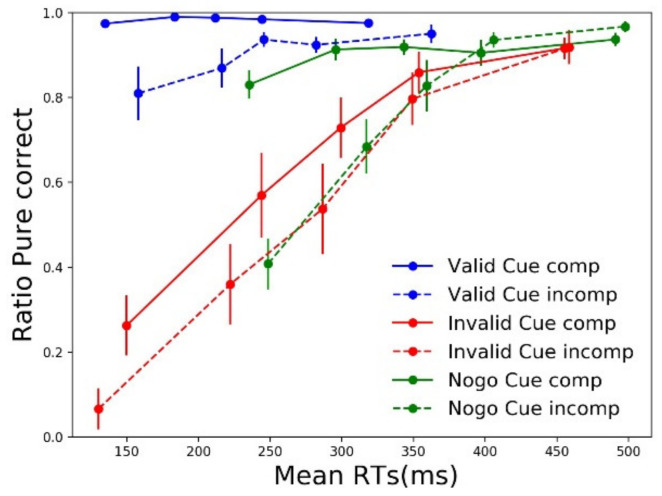
Conditional incorrect accuracy function. The ratio of correct responses is plotted as a function of RTs, divided in quintiles separately for each cue and compatibility condition. Error bars represent the standard error.

Conditional accuracy functions (CAFs), as reported in Section 3.2.2, are based on overt errors. Those overt errors, however, only reflect uncorrected incorrect response activations. To better assess the dynamics of automatic incorrect response activation, irrespective of its later correction or not, one can compute conditional incorrect activation functions—CIAFs (see Section 2)—by taking into account all (first) incorrect response activations. This measure has been effectively used to assess inhibitory control deficits in patients affected by Parkinson’s Disease [52] and attention deficit hyperactivity disorder [50]. In the present context, CAF suggested that, after a valid cue, no more automatic activation by the stimulus position occurred. CIAF will provide a different view. Figure 6 plots the resulting CIAF for each cue and compatibility condition.

Since a full ANOVA could not be performed, and we were interested in testing compatibility effects separately for each cue type at the fastest RT bin, we adopted Wilcoxon tests to run pairwise comparisons. Partial errors were triggered more frequently in incompatible compared to compatible trials for all three cue types (valid: W = −3.170, *p* = 0.002; NoGo: W = −3.516, *p* < 0.001; invalid: W = −2.666, *p* = 0.008). EMG-based analyses reveal that the incorrect effector was activated by the incompatible stimulus position in a high number of NoGo cue trials, although a minority of those resulted in full errors. While valid cues significantly reduced the number of errors, even for the fastest incompatible responses (first dot of blue lines in Figure 4), the incorrect effector was still subliminally activated in incompatible trials, resulting in partial errors (first dot of blue dashed line in Figure 6).

#### 3.3.5. Effect of Motor Preparation on EMG Categories

**Figure 7:**
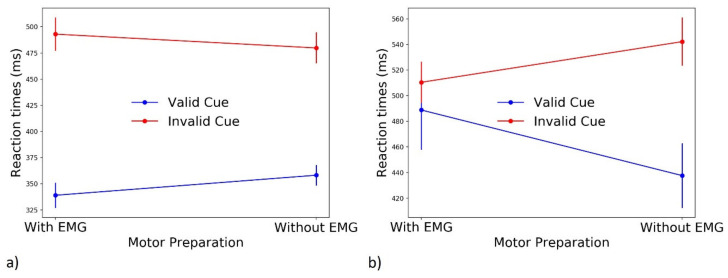
Average RTs for valid and invalid cue trials are plotted for pure correct (**a**) and partial errors (**b**). Error bars represent the standard error.

Behaviorally correct responses include both PC and IC trials. First, we replicated previous results showing that IC trials result in significantly slower RTs: indeed, while the latency of the incorrect EMG of the partial error trials is rather fast, the (correct) RT of these trials is much longer than the Pure-Correct ones. We also previously showed that motor preparation positively affects RTs in valid cue trials. While we failed to find an effect in invalid cue trials, dividing PC from IC trials could provide an explanation for this null effect. However, since the number of participants producing partial errors with and without motor preparation activity was variable, we could not perform a full ANOVA. Separate Student’s *t*-tests were therefore used to compare, for each trial type (IC, PC) and Go cue type (valid, invalid), average RTs calculated in trials with vs. without preparatory EMG activity. For valid cue PC trials, we found a beneficial effect of motor preparation in response speed (339 ± 48 ms with EMG, 358 ± 39 ms without EMG; t (15) = −2.711, *p* = 0.016). The same analysis in IC trials was not statistically significant (483 ± 99 ms with EMG, 438 ± 76 ms without EMG; t (8) = 1.135, *p* = 0.3). Conversely, for invalid cue PC trials, we did not find an effect of motor preparation (493 ± 64 ms with EMG, 480 ± 59 without EMG; t (15) = 1.172, *p* = 0.26), but we found a significant effect in IC trials (510 ± 63 ms with EMG, 542 ± 75 ms without EMG; t (14) = −2.865, *p* = 0.012). The effects of this double dissociation are displayed in Figure 7. These results suggest that the previously described beneficial effect of preparatory motor activity triggered by valid cues on RTs is mostly due to PC trials (blue line in Figure 7a). Conversely, while we did not find effects considering all correct responses, a positive effect of motor preparation following invalid cues is present in partial errors (red line in Figure 7b). This effect is not due to differences in the number of partial errors included in the analysis, since they were equally frequent in invalid cue trials with and without motor preparation (Wilcoxon test: W = 54, *p* = 0.73). While this result may seem counterintuitive, partial error’s onset latencies were found to differ between invalid cue IC trials with and without preparatory motor activity (152 ± 34 ms and 177 ± 35 ms, respectively; t (14) = −3.905, *p* = 0.001). This, in turn, suggests that when participants overtly prepared the (incorrectly) cue-instructed effector (red dot in Figure 7b “with EMG”), they were more ready to respond to the presentation of the target. IC RTs are composed of the latency of the partial error, the “correction time”, from partial error onset to the onset of the correct response, and the “motor time”, that is the time between EMG onset of the correct response and the button press. While we found an effect of partial errors’ onset latency, neither correction times (t (14) = −0.148, *p* = 0.88) nor motor times (t (14) = −0.83, *p* = 0.42) were impacted by motor preparation. Therefore, while the first EMG activity following target onset is correct in valid cue trials, resulting in faster PC RTs, in invalid cue trials, this EMG response is instead a partial error. Since the onset of partial errors following preparatory EMG activity occurs earlier, global RTs are also faster compared to invalid cue IC trials without preparatory EMG activity. Motor preparation can, therefore, be considered an objective index of participants’ readiness to respond and, when errors are avoided, it is always beneficial to response speed.

Finally, to investigate whether motor preparation impacted the compatibility effect in invalid cue IC trials, we ran a repeated measures ANOVA with factor compatibility (compatible vs. incompatible trials) and motor preparation (with vs. without EMG activity). We replicated the previous motor preparation effect (F (1, 14) = 9.396, *p* = 0.008, η^2^ = 0.402), without a significant main effect of compatibility (F (1, 14) = 0.286, *p* = 0.6, η^2^ = 0.02) nor an interaction (F (1, 14) = 2.514, *p* = 0.135, η^2^ = 0.152). Invalid cue IC trials were faster when participants prepared the cue-coherent effector before target onset in both compatible and incompatible trials.

## 4. Discussion

In this study, we investigated the relationship between motor preparation and both proactive and reactive inhibitory mechanisms. To this aim, we adopted a threefold strategy: First, we developed an ad hoc cued Go/NoGo Simon paradigm; second, beyond mean performance, the use of distributional analyses allowed us to better characterize the dynamics of the processes of interest; third, recording EMG activity of the muscles involved in the task allowed us to reveal subliminal response activations not observable in behavior (preparatory activities and partial errors). The paradigm allowed us to (i) induce conditions requiring both proactive and reactive action inhibition, (ii) motivate participants to prepare the cued response before target onset, and (iii) induce relatively frequent partial errors.

We induced proactive inhibition by (i) adopting a Go/NoGo paradigm in which, in every trial, there was uncertainty as to whether a response might have to be executed or not (generating a “preparing to stop” process); (ii) introducing a fixed delay between the moment in which participants could plan the response (cue onset) and its execution (following a Go target), allowing participants to predict target onset. Since targets required the cued response twice more frequently than any other option (see Figure 1a), and since Go trials were overall twice as frequent as NoGo trials, participants did follow cue instructions, as suggested by EMG data collected in the foreperiod (Supplementary Figure S3).

In NoGo cue trials, participants did not expect to execute a response, as indicated by the virtually absent motor preparation in the foreperiod. Since no motor preparation occurred, impulse control mechanisms were likely not activated. However, while motor preparation and execution mechanisms could have simply been delayed, the slow RTs rather suggest the presence of a proactive inhibitory mechanism. Indeed, cue validity induces both costs and benefits (as indicated by the EMG data). Had the participant not used the NoGo cue (hence acting as a “neutral” cue), their performance should lie in between the valid and invalid condition. The fact that NoGo cue Go RTs are equivalent to invalid cue Go trials’ RTs indicates that a cost is present in the former condition and, hence, that the to-be-performed response is in an “inhibited” state, as following an invalid cue. A NoGo cue-enhanced “preparing to stop” process explains the longer RTs we found in NoGo cue Go trials, similarly to the slow no-stop RTs found in the context of the SSP [3,9]. In line with this interpretation, participants committed very few (fast) errors (Figure 2b and Supplementary Figure S2), confirming that they did not expect to respond and did not plan any action in advance, likely not needing impulse control mechanisms. A limitation of this study is the absence of an uncued condition in which the simple impact of the Go/NoGo paradigm on proactive inhibition as “preparing to stop” process could be tested and compared to NoGo cue trials.

In both valid and invalid cue trials, participants had to wait for target onset to either execute a button press or inhibit (reactively) the planned response. These two conditions were, therefore, equal in terms of proactive inhibition mechanisms. Nonetheless, task performance was greatly impacted by cue validity. Valid cues resulted in the best performance, with fast RTs (Figure 2a) and rare full (Figure 2b) and partial errors (blue lines in Figure 5). Moreover, when partial errors did occur on valid cue trials, it was mostly in incompatible trials being triggered by the task-irrelevant stimulus location (dashed blue lines in Figure 5 and Figure 6). Conversely, invalid cues not only elicited slower RTs (Figure 2a), more (fast) errors (Figure 2b and Supplementary Figure S2), and partial errors (red lines in Figure 5), but the latter also occurred quite frequently in compatible trials (filled red lines in Figure 5 and Figure 6), especially at short latencies. This result suggests that partial errors occurring in compatible trials are due to the preceding cue-triggered motor preparation, which turns out to be the incorrect one in invalid cue trials. Previous studies have found that compatible trials elicit partial errors, though to a smaller extent [36,37]. While expectations regarding target identity were intentionally manipulated through cue validity here, we suggest that preparatory motor activities in the pre-target period can be used to predict whether partial errors will occur in conflict-free trials. While we do not claim that overt EMG activity is the necessary signature of an ongoing motor preparation mechanism, it surely signals a strongly prepared response, closer to the threshold for action execution. In line with this hypothesis, when preparatory EMG activity in the incorrectly cued effector was found late in the foreperiod of invalid cue trials, accuracy was lower (Supplementary Figure S5). Moreover, virtually zero valid and NoGo cue ICs found in compatible trials contained preparatory motor activity. Such rare ICs might, therefore, be due to temporary lapses of attention. Conversely, more than half of the invalid cue ICs found in compatible trials contained preparatory EMG activity. Future studies should investigate whether brain circuits responsible for proactive inhibition (see [53] for a review) are differentially modulated in IC trials in which the incorrect effector was prepared before target appearance.

Motor preparation has a beneficial effect on performance. While this effect could be expected following valid cues (especially PC trials, see Figure 7a), interestingly, we found a similar effect in invalid cue IC trials (Figure 7b). Since invalid cues instructed participants to prepare a contralateral response to that ultimately instructed by target identity, it could seem counterintuitive that, when cue-triggered motor preparation is stronger, RTs should be faster. However, we also found that the latency of partial errors is shorter in invalid cue IC trials with preparatory EMG activity, suggesting that, the earlier a partial error occurs, the sooner reactive inhibition can correct for it, generating overall faster RTs.

For Go cues that turned out to be invalid, participants likely withheld, through proactive inhibition, the incorrectly prepared effector (e.g., left hand for a square cue) up to target onset (e.g., triangle). When the target appears, proactive inhibition is thought to be released [1,29,54] and reactive action inhibition is then needed to prevent this incorrect activation to becoming an error. Reactive inhibition was able to prevent errors on an average of 80% in invalid cue compatible trials (e.g., square cue followed but a triangle presented on the right, see CAF in Figure 4), in which both stimulus identity and position indicated to participants that a response was to be executed with the contralateral effector (left hand, in this example) to the planned response. Conversely, whenever the target was displayed at the incompatible location (e.g., triangle presented on the left), the stimulus’ position reinforced the incorrectly prepared (left hand) response, resulting in even more frequent errors (average accuracy 67%). These results suggest that informative cues allow the investigation of the interaction between proactive and reactive action inhibition. However, the analysis of overt responses remains limited. In fact, while CAF data suggest that no automatic activation of the incorrect effector occurred in valid cue incompatible trials (Figure 4), EMG-based distributional analysis of covert incorrect activations (CIAF, Figure 6) suggests that an automatic capture in such trials does occur, but that virtually all subliminally incorrect response activations are corrected before becoming overt errors.

While average RTs are not able to disentangle NoGo cue and invalid cue trials (Figure 2a), distributional analyses of RTs (Figure 3 and Supplementary Figure S2) allow for a better investigation of their response patterns. NoGo cues induce a typical reduction in compatibility effects as RTs increase [33,49]. According to the activation–suppression hypothesis [39], the incorrect automatic activation of the incorrect effector, driven by a stimulus’ location in incompatible trials of the Simon task, needs time to be inhibited. Therefore, the compatibility effect is expected to be greater for faster responses, in which this online inhibitory mechanism is not yet completed, and incompatible RTs are much slower than compatible RTs. This effect was likely boosted by the temporal predictability of target onset, due to the constant foreperiod duration [55], resulting in a steep negative-going slope for NoGo cue trials. Informative go cues, together with target onset’s temporal predictability, allowed participants to prepare a response in advance and be ready to respond at target onset. In invalid cue trials, the incongruency between cue and target identity also slowed down responses to compatible trials, therefore reducing the compatibility effect already at fast RTs. Conversely, following a valid cue, the correct effector was likely very close to the threshold for action execution at target onset, as confirmed by the faster RTs (Figure 2a). However, an inter-trial and inter-subjects’ variability also exists as to how much participants followed cue instruction. Therefore, one possibility is that when participants were ready to respond at target onset, following a valid cue, the conflicting information of the incompatible stimulus location had little room to slow down a response that was already close to the threshold for execution (see first dot of dashed blue line in Figure 3 and Figure S2). On the contrary, when participants were less ready to respond, a stronger compatibility effect arose. In line with this interpretation, at similar RTs, valid and NoGo cues induced a similar compatibility effect (bins 1 and 2 for valid cues and bins 4 and 5 for NoGo cues).

## 5. Conclusions

In conclusion, we provided evidence that impulse control mechanisms help to prevent errors. When correct responses are considered, motor preparation, be it correct or incorrect (requiring reactive inhibitory mechanisms), has a beneficial effect on response speed. While we replicated previously found slow RTs in task conditions, supposedly activating proactive inhibition, we suggest that they may result from absent motor preparation or an increased general “preparing to stop” process, but not from impulse control mechanisms. For task conditions that equally involved proactive inhibition mechanisms, motor preparation played a key role in shaping the behavior and efficiency with which reactive action inhibition prevented covert incorrect activations from becoming full errors. Specifically, subliminal incorrect activations in conflict-free conditions are likely due to incorrectly planned responses, and peripheric or cortico-spinal measures of preparatory motor activity should be used to prevent or predict them.

## Data Availability

Data available on request.

## Supplementary Materials

The following are available online at https://www.mdpi.com/article/10.3390/brainsci11060680/s1, Figure S1: Task structure: examples of valid and NoGo cue trials, Figure S2: Distribution data for correct and incorrect responses, Figure S3: Cue-triggered motor preparation, Figure S4: Valid cue trials: correlation between the cumulative amplitude of correct cue-triggered EMG activity measured in the cue-target period and RTs, Figure S5: Invalid cue trials: correlation between the latency of cue-triggered EMG activity measured in the cue-target period and accuracy.
