# Supplementary material for: Preparing to React: A Behavioral Study on the Interplay between Proactive and Reactive Action Inhibition
Source: Brain Sci. 2021 May 22;11(6):680. doi: 10.3390/brainsci11060680 (PMC8224560; doi:10.3390/brainsci11060680)
Supplement: Supplementary file 1 [file brainsci-11-00680-s001.zip › Supplementary material.pdf]

Supplementary material

Task structure: examples of valid and NoGo cue trials

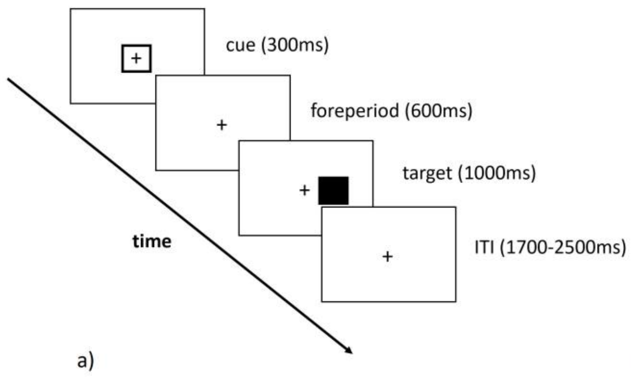

S1: Examples of valid cue (S1a) and NoGo cue (S1b) trials are displayed. Following a 300ms-long presentation of a central cue and a 600ms-long foreperiod, a lateralized target is displayed on the ipsi- or contralateral side of the required response, distinguishing compatible and incompatible trials. In the valid cue example trial displayed in S1a, the square cue instructs (half of the) participants to prepare a left-hand button press. The same shape is presented as target, instructing participants to execute a left-hand response. Since the stimulus location (left) is ipsilateral to the side of the instructed action, the trial is compatible. In the NoGo cue example trial displayed in S1b, a cercle (NoGo cue) instructs participants that they will mostly likely not have to respond. A square target, instructing participants to respond with the left hand, is presented on the right side of the screen (incompatible trial). Following a variable inter-trial-interval (ITI), a new trial starts. Colors are inverted for displaying purposes.

Distribution data for correct and incorrect responses

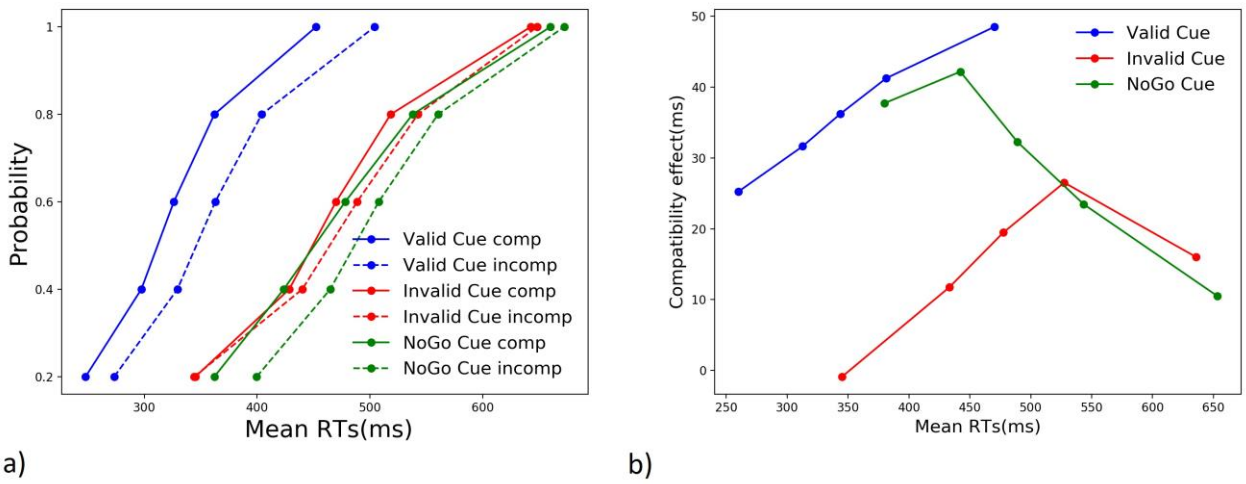

S2: Cumulative distribution function of RTs (S2a). Delta plots created including both correct and incorrect responses (S2b). The compatibility effect (incompatible RTs – compatible RTs) is plotted as a function of RTs.

Cue-triggered motor preparation

To test whether participants followed the task requirements, that is preparing the response effector coherently with the cue, we calculated the percentage of trials in which any significant EMG activity could be detected in the cue-target period. Such preparatory activity was considered correct (Corr_Go) if it occurred on the effector instructed by the Go cue (right hand following triangle cue for half of the participants and left hand following triangle cue for the other half; the opposite for the square cue) and incorrect otherwise (Err_Go). Since the NoGo cue (circle) instructed participants to not prepare any response, any EMG activity was considered erroneous (Err_NoGo). As shown in supplementary figure S1, preparatory EMG activity on Go cue trials is more often correct (29%) than not (1,48%). Furthermore, participant very rarely prepared a response following a NoGo cue (3,88%). These data suggest that participants followed task instructions.

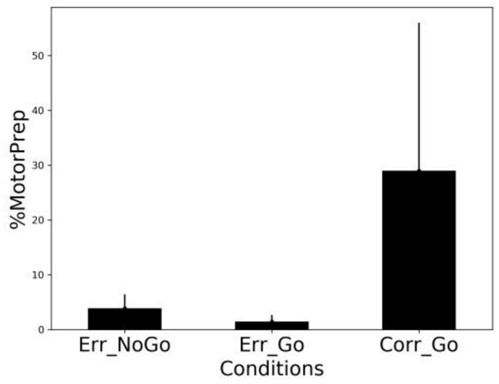

S3: Average percentage of detected motor preparation that occurred after a NoGo cue (Err_NoGo) and after a Go cue (wrong motor preparation for Err_Go, correct motor preparation for Corr_Go). Error bars indicate standard deviation.

Valid cue trials: correlation between the cumulative amplitude of correct cue-triggered EMG activity measured in the cue-target period and RTs

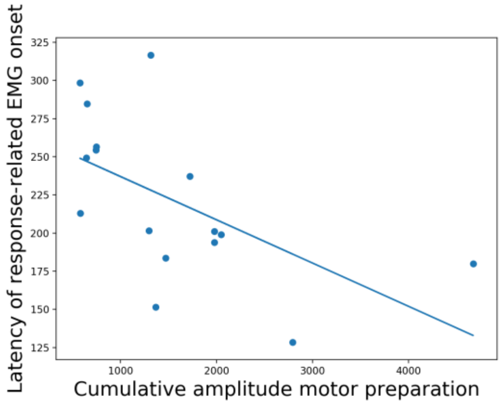

S4: The scatterplot shows the relationship between the amplitude of preparatory motor activity and RTs for valid cue trials. The amplitude of motor preparation was calculated as the cumulative amplitude of EMG signal, measured from the cue-instructed effector, in the cue-target period. For RTs, the latency of the response-related EMG onset was used. The interpolation line shows the direction of the correlation between the two variables.

Invalid cue trials: correlation between the latency of cue-triggered EMG activity measured in the cue-target period and accuracy

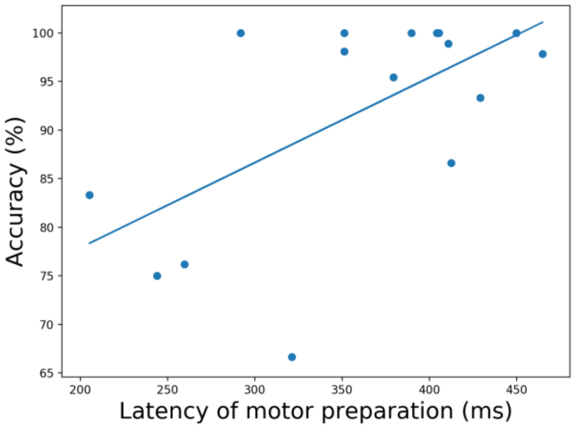

S5: The scatterplot shows the relationship between the latency of preparatory motor activity and accuracy for invalid cue trials. The latency of motor preparation was calculated with respect to the latency of target onset, so that greater values correspond to an earlier motor preparation. Accuracy data is represented as percentage of correct responses. The interpolation line shows the direction of the correlation between the two variables.
